# A case of hypertrophic cardiomyopathy presenting with high-degree atrioventricular block

**DOI:** 10.1097/MD.0000000000046557

**Published:** 2025-12-12

**Authors:** Yan Gu, Fuzhong Chen, Chao Zhang

**Affiliations:** a Department of Cardiovascular Medicine, Zhangjiagang Second People’s Hospital, Suzhou, China; b Department of Cardiovascular Medicine, The Second Affiliated Hospital of Nanjing Medical University, Nanjing, China; c Department of Geriatric Medicine, Bengbu Hospital of Traditional Chinese Medicine, Bengbu, China.

**Keywords:** gene variant, high-degree atrioventricular block, hypertrophic cardiomyopathy, pacemaker implantation

## Abstract

**Rationale:**

Hypertrophic cardiomyopathy (HCM) with high-degree atrioventricular (AV) block is rarely reported in China. This case is unique for a 35-year-old female with progressive high-degree AV block and dual heterozygous variants (MYH7 c.748A > T, DES c.553G > A), supplementing HCM’s clinical-genetic spectrum.

**Patient concerns:**

The patient had 11-year post-exertional chest pain (aggravated recently), family history of sudden cardiac death, hypotension (87/46 mm Hg), bradycardia (50 bpm). ECG progressed to complete heart block; echocardiography showed left atrial enlargement, septal thickening, apical trabecular hyperplasia; cardiac MRI revealed septal fibrosis. Pro-BNP (846 ng/L) and troponin T (12 ng/L) were elevated. Genetic testing found MYH7 (highly suspected pathogenic) and DES (suspected pathogenic) variants; family screening identified 2 affected individuals.

**Diagnoses:**

HCM, high-degree AV block, left atrial enlargement.

**Interventions:**

Dual-chamber pacemaker implantation, metoprolol + spironolactone.

**Outcomes:**

Symptom relief, no perioperative complications; long-term follow-up initiated.

**Lessons:**

HCM with high-degree AV block needs genetic testing; pacemaker implantation and long-term monitoring are key for preventing sudden cardiac death. Family screening is vital for risk stratification.

## 1. Introduction

**Figure 1.**
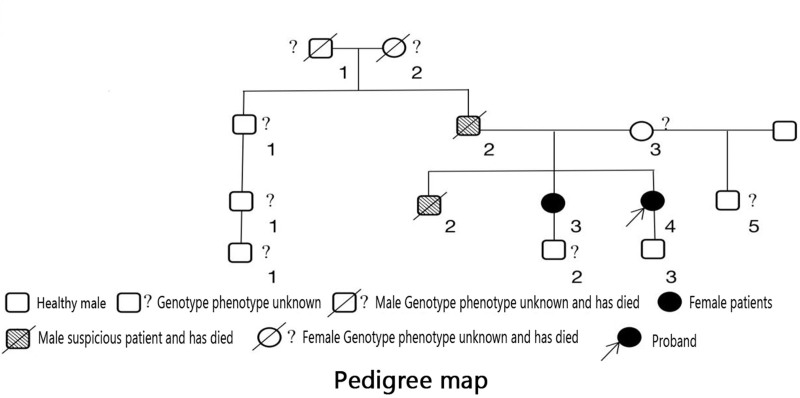
The family pedigree chart.

Hypertrophic cardiomyopathy (HCM) is characterized by left ventricular myocardial hypertrophy and is primarily attributed to genetic variants in sarcomere protein-encoding genes (or genes associated with sarcomere proteins) or genetic causes of unknown etiology. Other causes of left ventricular hypertrophy, including those resulting from clear evidence of other cardiac, systemic, or metabolic diseases, must be ruled out.^[1]^ Currently, it is believed that HCM is mainly caused by gene variant, and it is estimated that there are at least 30 pathogenic genes with 1500 variants. Among various genetic alterations, missense variants are the most prevalent type. Significantly, the most commonly observed and highly relevant variant lies in the gene encoding sarco-myoglobin. MYH7 and MYBPC3 gene variants are prevalent in over 70% of patients with hypertrophic cardiomyopathy (HCM). The majority of HCM patients exhibit autosomal dominant inheritance, and approximately 50% of them can have definite pathogenic variants of the gene encoding myostatin identified.^[2]^ However, cases of HCM complicated with high-grade atrioventricular block have been rarely reported in China. This article reports one such case and includes a family investigation (Fig. 1).

## 2. Case report

**Figure 2.**
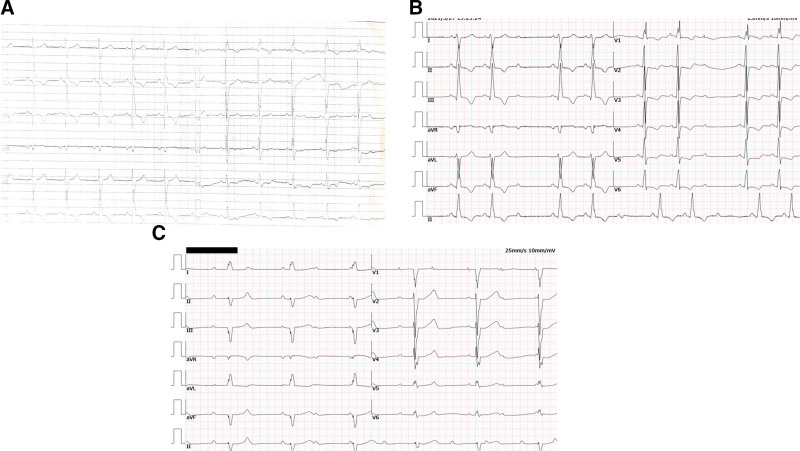
ECG changes of the patient in the last 2 years. (A) ECG in 2019: sinus rhythm, CRBB; (B) ECG on admission: CRBB, second degree atrioventricular block with prolonged Q-T interval; (C) during hospitalization: CRBB, third degree atrioventricular block with prolonged Q-T interval. CRBB = complete right bundle branch block, ECG = electrocardiogram.

**Figure 3.**
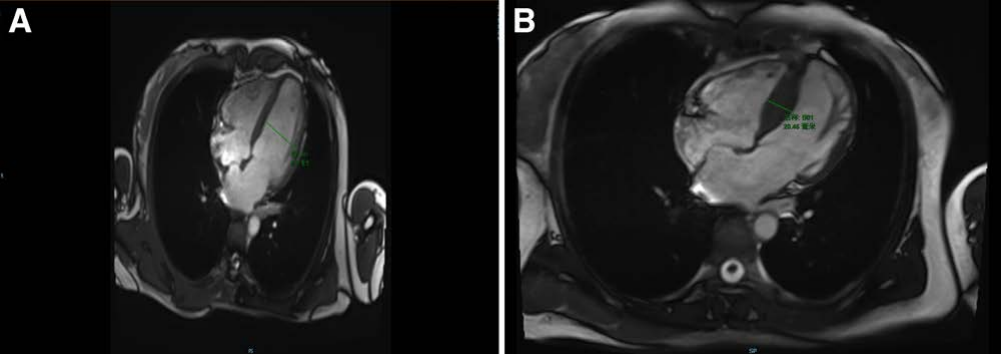
Cardiac MR of the proband and her sister. (A) Cardiac MRI of the proband’s heart: left ventricular systolic function was slightly weakened, with patellar fibrosis of the ventricular septum and lateral wall; (B) cardiac MRI of the second-generation (II3): thickening of interventricular septum (20 mm), the anterior and inferior wall of the left ventricle with left ventricular myocardial fibrosis and focal myocardial edema. MRI = magnetic resonance imaging.

The index patient, a 35-year-old female, was admitted to hospital because of chest pain after exercise for 11 years, aggravated with chest tightness for 1 week. The patient had previously experienced chest pain after vigorous exercise. However, in the past week, she experienced chest pain and discomfort after climbing 3 flights of stairs, which could be relieved by resting in a squatting position for more than 10 minutes. Her father and brother had a history of sudden cardiac death (SCD). On admission, her vital signs showed a blood pressure of 87/46 mm Hg, a heart rate of 50 beats per minute with irregular rhythm, and no murmurs were detected on auscultation. Echocardiography performed over the past 2 years had shown a trend of thinning in the interventricular septum (Table 1). The electrocardiogram showed significantly changes during the past 2 years. In 2019, the ECG showed normal sinus rhythm, normal PR interval (PR), complete right bundle branch block (CRBB) and likely left posterior fascicular block (Fig. 2A), however, the ECG on admission showed sinus rhythm, 3:2 Mobitz II second degree AV block, CRBB and RBBB and left posterior fascicular block (Fig. 2B). During hospitalization, sinus rhythm with complete heart block, and slow ventricular escape rhythm with LBBB morphology, suggesting the escape arises from the right bundle branch (Fig. 2C). Routine laboratory tests, including complete blood count, urinalysis, stool examination, biochemical profile, electrolytes, and immunological assays were all within normal limits. Her Pro-BNP was 846 ng/L and the cardiac troponin T was 12 ng/L. Two-dimensional echocardiography revealed left atrial enlargement with an inner diameter of 45 mm, nonuniform enhancement of the interventricular septum with a mid-segment thickness of 12 mm, and the left ventricular posterior wall thickness of 6 mm, resulting in a septal-to-posterior wall thickness ratio of 2:1. The basal segment of the left ventricular posterior wall had thinner myocardium, measuring approximately 2 to 3 mm, with increased echogenicity and local outward bulging, exhibiting significantly reduced pulsation. The myocardium in the dense layer of the left ventricular apex was thin, and the muscle trabeculae were abundant, forming a deep recess, low blood flow in the recess, and the myocardial beat in the left ventricular apex was weakened. Strain echocardiography indicated reduced myocardial strain at multiple locations in the left ventricle (Table 1). Cardiac magnetic resonance imaging (MRI) (Fig. 3A) showed slightly reduced left ventricular systolic function, patchy fibrosis in the interventricular septum and lateral wall, and nodular thickening of the middle segment of the interventricular septum in the left ventricle. There was minimal pericardial effusion. The patient received dual-chamber pacing (DDD) implantation and was prescribed with metoprolol and spironolactone postoperatively.

**Table 1:** ECG and echocardiographic data of patients in the recent 2 years.

	2019	2020	2021	One month after pacemaker implantation
2DE				
IVS (mm)	15	12	12	12
LVPW (mm)	10	10	6	5
LAD (mm)	32	33	45	36
LVEd (mm)	44	40	51	51
EF%	58	60	56	56
2D-STI				
LVEF% (Simpson)		50		50
GLPS-LAX (%)		−15.36		−17.34
GLPS-A4C (%)		−16.62		−15.35
GLPS-A2C (%)		−13.15		−12.65
GLPS-Avg (%)		−15.04		−15.11

2DE = 2-dimensional echocardiography, 2D-STI = 2 dimension-strain image, GLPS-A2C = global longitudinal peak strain of Apical 2 chamber, GLPS-A4C = global longitudinal peak strain of Apical 4 chamber, GLPS-Avg = global longitudinal peak strain of Average, GLPS-LAX = global longitudinal peak strain of Long axis, IVS = Interventricular septum, LAD = left atrial diameter, LVDd = left ventricular end diastolic diameter, LVEF = left ventricular ejection fraction, LVPW = left ventricular posterior wall.

**Figure 4.**
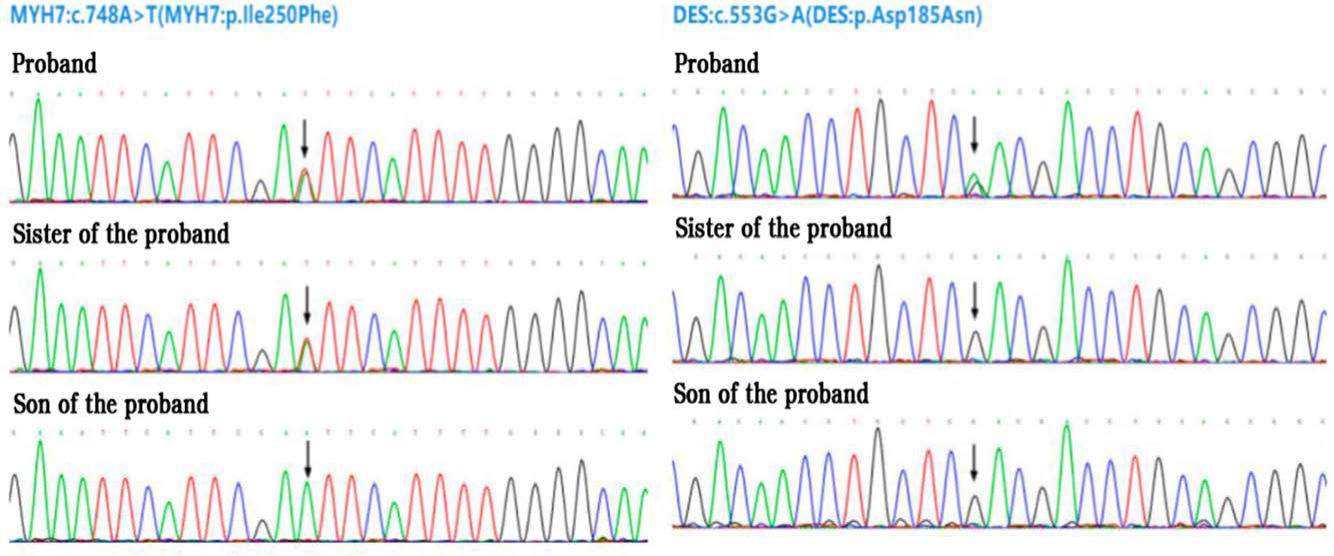
The sequencing data of the proband, her sister and her son.

Family investigation: 3 generations, 13 individuals, with 2 affected patients, both of whom are female and alive. Two individuals with suspected disease, both male, with a family history of SCD in the index patient’s father and brother (Fig. 1). Examination of the second-generation (II3) revealed a grade II systolic murmur along the left sternal border in the fourth intercostal space. Echocardiography showed spindle-shaped hypertrophy of the interventricular septum, with the thickest segment measuring approximately 14 mm, and apical segment myocardial hypertrophy of approximately 9 to 10 mm. The ratio of interventricular septum thickness to left ventricular posterior wall thickness was 1.3:1, and the peak velocity of the left ventricular outflow tract was 0.9 m/s at rest, which increased to 1.5 m/s after squatting 20 times at the bedside. Cardiac MRI (Fig. 3B) suggested a diagnosis of nonobstructive hypertrophic cardiomyopathy, with thickening of the left anterior ventricular wall, interventricular septum, and inferior wall myocardium, with the thickest part of the interventricular septum measuring approximately 20.5 mm. There was associated fibrosis and focal myocardial edema in the left ventricular myocardium, along with left atrial enlargement. The diagnosis was nonobstructive hypertrophic cardiomyopathy. Using NGS + Sanger sequencing, the index patient was found to carry the pathogenic variant MYH7 c.748A > T (MYH7:p.Ile250Phe het) associated with HCM and also carried a rare variant DES c.553G > A (DES:p.Asp185Asn het) – a variant reported to be potentially associated with autosomal dominant dilated cardiomyopathy (DCM) type 1I,^[3]^ though its causal role in DCM remains unconfirmed. The second-generation individual (II3) carried the pathogenic variant MYH7 c.748A > T (MYH7:p.Ile250Phe het) associated with HCM but did not carry any pathogenic genes associated with DCM. The third-generation individual (III3) did not carry any relevant genetic variants (Fig. 4).

## 3. Discussion

HCM is an autosomal dominant genetic disease, which is the main cause of sudden death in adolescents and athletes at home and abroad. The prevalence of HCM in the global general population is at least 0.2%, and the prevalence of HCM in Chinese adults is 80/100,000,^[4,5]^ which deserves attention. SCD is more commonly observed in young patients aged 10 to 35 years, and while the risk of SCD decreases with age, it never completely disappears. In patients treated at tertiary medical centers, the annual mortality rate for HCM ranges from 2% to 4%, with SCD being one of the most common causes.^[6]^In a European multicenter retrospective cohort study, SCD was the most common cause of death in patients with HCM, although it had the lowest incidence of HCM-related complications (about 1%). In young patients (16–20 years old), SCD accounts for up to 80% of deaths.^[7]^ A recent large meta-study showed that the prevalence of contemporary HCM-associated SCD has decreased by 50% compared to pre-2000, and the prevalence of contemporary HCM-associated SCD is 0.32%/ year, but the risk is still highest in young people.^[8]^ During lifetime follow-up of HCM, it was found that family history of SCD was the most important risk factor for the occurrence of SCD. When there were ≥ 2 sudden death events in the family, the survival rate of HCM patients would decrease rapidly, which was particularly necessary for risk stratification and preventive treatment of SCD.^[9]^ At present, HCM has a high prevalence rate, high mortality, complex etiology and pathogenic mechanism, and diverse clinical phenotypes. Therefore, it is still very difficult to accurately diagnose and treat HCM.

The patient in our case carries a heterozygous missense variant MYH7:c.748A > T (MYH7:p.Ile250Phe het). The MYH7 gene encodes the beta-heavy chain subunit of cardiac myosin and is primarily expressed in the ventricles. HCM type 1 (autosomal dominant, AD) is characterized by asymmetric ventricular septal hypertrophy, apical hypertrophy (in some patients), basal narrowing of the aorta, hypertrophic cardiomyopathy, presystolic gallop rhythm, palpitations, arrhythmias, congestive heart failure, and sudden death. Missense variants near this locus, such as c.761C > A (p.Ala254Glu), c.755T > G (p.Phe252Cys), c.746G > A (p.Arg249Gln), and c.743 T > C (p.Ile248Thr), have been repeatedly confirmed as pathogenic or potentially pathogenic variants associated with HCM. Although the HGMD database does not list this variant, other missense variants in the same position, such as c.748 A > G (p.Ile250Val), have been proven pathogenic in HCM. Currently, this variant is rare, with a frequency of 0 in local databases. According to the analysis software for pathogenic variants in inherited cardiomyopathy by “BGI Genomics,” this variant is predicted to affect protein function. However, due to the lack of evidence for familial linkage and functional studies, this variant is classified as a highly suspected pathogenic variant for HCM type 1 (Class B). Further molecular biological research is needed to establish the causal relationship between this variant and HCM.

An interesting aspect is that the patient also carries a rare variant, DES:c.553G > A (DES:p.Asp185Asn het), in the DES gene. The DES gene encodes desmin, a muscle-specific intermediate filament protein that forms stable filamentous networks in the cytoplasm, serving as a connecting structure between myofibrils and the cell membrane. Related diseases include DCM type 1 (autosomal dominant, AD), characterized by dilated cardiomyopathy, restrictive cardiomyopathy, rare cases of hypertrophic cardiomyopathy, cardiac enlargement, chronic heart failure, dual atrial enlargement, atrial fibrillation, atrial flutter, partial or complete atrioventricular block, right bundle branch block, diffuse left ventricular hypokinesis, reduced left ventricular ejection fraction, right ventricular dilation, circumferential fibrosis (late stage), and visible Desmin aggregation in ventricular myocytes.^[3]^ In this case, the patient exhibits clinical features of both hypertrophic and dilated cardiomyopathy, with involvement of the conduction system, leading to high-degree atrioventricular block, resembling the presentation of DCM type 1 (AD). However, due to the lack of evidence for familial linkage and functional studies, this variant is classified as a suspected pathogenic variant (class C1) for DCM. Further molecular biological research is needed to establish the causal relationship between this variant and DCM. Given the presence of high-degree atrioventricular block in this patient, a dual-chamber pacemaker was implanted. For such patients, long-term follow-up is essential, which will provide guidance for the patient’s future treatment. During the follow-up period, close monitoring of ECG changes is necessary for such patients. Changes in the ECG can provide a reference for determining whether there is impairment of myocardial electrophysiological function. A study that evaluates ventricular polarization in noncompaction cardiomyopathy (NCCM) using ECG offers important references for interpreting such electrophysiological manifestations, as genetic cardiomyopathies (including hypertrophic cardiomyopathy (HCM) and NCCM) often associate structural lesions with ECG abnormalities.^[10]^ Additionally, machine learning-assisted ECG analysis for myocardial fibrosis in HCM further confirms the value of ECG in the comprehensive assessment of HCM.^[11]^

In summary, for patients with hypertrophic cardiomyopathy combined with high-degree atrioventricular block, pacemaker implantation is recommended to prevent sudden death. Prognosis for this patient population is generally poor, with a high incidence of cardiovascular events. Long-term follow-up with home-based continuous cardiac monitoring and 2-dimensional echocardiography is crucial for the clinical management of these patients.

## 4. Take home messages

Hypertrophic cardiomyopathy with high-degree atrioventricular block is a complex condition requiring comprehensive assessment including genetic testing. Implantation of a dual-chamber pacemaker and long-term follow-up are crucial for the management and prognosis of such patients.

## Acknowledgments

We sincerely acknowledge the support for this study from the 2024 Scientific Research Project on Traditional Chinese Medicine of Anhui Provincial Association of Traditional Chinese Medicine (Project No. 2024ZYYXH075).

## Author contributions

**Visualization:** Fuzhong Chen.

**Writing – original draft:** Yan Gu, Chao Zhang.
